# High-intensity compared to moderate-intensity training for exercise initiation, enjoyment, adherence, and intentions: an intervention study

**DOI:** 10.1186/1471-2458-14-789

**Published:** 2014-08-03

**Authors:** Katie M Heinrich, Pratik M Patel, Joshua L O’Neal, Bryan S Heinrich

**Affiliations:** Department of Kinesiology, Kansas State University, Functional Intensity Training Lab, 1A Natatorium, Manhattan, KS 66506 USA; Department of Radiology, Michigan State University, East Lansing, MI USA

**Keywords:** Exercise, High-intensity, Functional movements, Moderate-intensity, Overweight, Obese, CrossFit, Enjoyment, Adherence, Intentions

## Abstract

**Background:**

Understanding exercise participation for overweight and obese adults is critical for preventing comorbid conditions. Group-based high-intensity functional training (HIFT) provides time-efficient aerobic and resistance exercise at self-selected intensity levels which can increase adherence; behavioral responses to HIFT are unknown. This study examined effects of HIFT as compared to moderate-intensity aerobic and resistance training (ART) on exercise initiation, enjoyment, adherence, and intentions.

**Methods:**

A stratified, randomized two-group pre-test posttest intervention was conducted for eight weeks in 2012 with analysis in 2013. Participants (n = 23) were stratified by median age (< or ≥ 28) and body mass index (BMI; < or ≥ 30.5). Participants were physically inactive with an average BMI of 31.1 ± 3.5 kg/m^2^, body fat percentage of 42.0 ± 7.4%, weight of 89.5 ± 14.2 kg, and ages 26.8 ± 5.9 years. Most participants were white, college educated, female, and married/engaged. Both groups completed 3 training sessions per week. The ART group completed 50 minutes of moderate aerobic exercise each session and full-body resistance training on two sessions per week. The HIFT group completed 60-minute sessions of CrossFit™ with actual workouts ranging from 5–30 minutes. Participants completed baseline and posttest questionnaires indicating reasons for exercise initiation (baseline), exercise enjoyment, and exercise intentions (posttest). Adherence was defined as completing 90% of exercise sessions. Daily workout times were recorded.

**Results:**

Participants provided mostly intrinsic reasons for exercise initiation. Eighteen participants adhered (ART = 9, 81.8%; HIFT = 9, 75%). HIFT dropouts (p = .012) and ART participants (p = .009) reported lower baseline exercise enjoyment than HIFT participants, although ART participants improved enjoyment at posttest (p = .005). More HIFT participants planned to continue the same exercise than ART participants (p = .002). No significant changes in BMI or body composition were found. Workouts were shorter for HIFT than ART (p < .001).

**Conclusions:**

HIFT participants spent significantly less time exercising per week, yet were able to maintain exercise enjoyment and were more likely to intend to continue. High-intensity exercise options should be included in public health interventions.

**Trial registration:**

ClinicalTrials.gov Identifier: http://NCT02185872. Registered 9 July 2014.

## Background

Few adults meet weekly guidelines of 150+ minutes of moderate-intensity or 75+ minutes of vigorous-intensity aerobic physical activity, and 2+ days resistance exercises [1, 2], often citing time as a barrier [3]. This is especially problematic for overweight and obese adults due to high risk for comorbid conditions [4]. For weight loss and control, at least double the weekly amount of moderate-intensity physical activity (>300 minutes) is recommended [2]. As overweight/obese adults are becoming the 'average’ U.S. adult population, understanding their low exercise participation and high dropout rates is critical [5].

High-intensity training (HIT) provides fitness and health improvements in less time per week than current guidelines [6, 7]. Although the intensity required might be intimidating, the reduced time requirement may be appealing to many adults, showing potential for higher rates of adherence. However, HIT often utilizes aerobic intervals which may not be sufficient; combined aerobic and resistance training among sedentary overweight and obese adults results in greater weight and fat loss and fitness improvements than each modality alone [8].

Extrinsic factors often motivate exercise initiation, yet facilitating intrinsic motivation is key for exercise adherence [9–11]. Adherence is also impacted by affective responses to exercise intensity, where enjoyment decreases as intensity increases, potentially contributing to high rates of physical inactivity [5, 12]. However, enjoyment rebounds post-exercise for HIT [5, 13, 14].

Group-based high-intensity functional training (HIFT) temporally combines aerobic and resistance exercises with focus on functional (multi-joint) movements, resulting in improvements to aerobic capacity and body composition [15]. HIFT intensity is self-selected by participants. This is important since self-selected intensity results in greater tolerance for HIT [5, 13], especially for previously inactive individuals [9].

Some concerns exist for HIFT, most notably the US Department of Defense and the American College of Sports Medicine have raised concerns for the potential of insufficient instruction in HIFT methods, need for scaling and training progressions, and the importance of monitoring participants for overtraining and injuries [16]. However, Army personnel participating in HIFT had slightly lower injury rates (12%) than those not participating in HIFT (i.e., exercising on their own or doing usual Army physical training; 14%), although higher body mass index (BMI) was associated with increased injury risk for HIFT participants [17]. A cross-sectional survey of HIFT participants found an injury rate of 3.1 injuries per 1000 hours trained, similar to those for weightlifting, powerlifting and gymnastics [18]. Otherwise, limited research exists for HIFT and behavioral responses to HIFT training are unknown. The purpose of this study was to examine effects of HIFT as compared to moderate-intensity aerobic and resistance training (ART) on exercise initiation, enjoyment, adherence, and intentions among physically inactive (<30 minutes per week) overweight and obese adults.

## Methods

### Design

Participants (n = 23) were recruited from Kansas State University and surrounding community to participate in a free exercise program which would consist of standard aerobic and resistance training or a CrossFit™ program. There was no racial or gender bias for participant selection. Participants were stratified on median age (< or ≥ 28) and BMI (<or ≥30.5) and randomized to eight weeks/24 sessions on non-consecutive days (Monday, Wednesday, and Friday) of HIFT or ART. Participants completed written informed consent and procedures were approved by the Kansas State University Institutional Review Board. The intervention was conducted in 2012 with analysis in 2013.

### Setting and participants

Participant characteristics included age (M = 26.8 ± 5.9 years), weight (M = 89.5 ± 14.2 kg), BMI (M = 31.1 ± 3.5 kg/m^2^), and body fat percentage (M = 42.0 ± 7.4). Race/ethnicity included white (n = 16), Hispanic (n = 3), Asian (n = 2), black (n = 1), and not specified (n = 1). Over half had a bachelor’s degree (60.9%), were female (56.5%) and were married/engaged (52.2%).

### Intervention

The ART intervention was designed to meet current physical activity recommendations and all sessions were supervised by an American Council on Exercise certified personal trainer [2]. Participants completed three exercise sessions per week on Mondays, Wednesdays, and Fridays. Participants first warmed up using aerobic exercise machines at their own discretion (time not recorded). They completed a total of 50 minutes of aerobic exercise on the machines (minimum of 10 minutes per machine) each session at 40-50% heart rate reserve (HRR) for weeks 1–4 and 50-60% HRR for weeks 5–8. Full-body resistance exercises were completed in about 20 minutes during the sessions on Mondays (i.e., bicep curls, military presses, lat pulldowns, and leg extensions) and Wednesdays (i.e., tricep pulldowns, bench presses, reverse leg curls, and seated leg presses). After establishing 1 repetition maximums (1RM) in week 1, participants completed three sets per lift for weeks 2–8 (i.e., weeks 2–3 = 50% 1RM, 15 reps; weeks 4–5 = 60% 1RM, 12 reps; weeks 6–7 = 70% 1RM, 10 reps; week 8 = 75% 1RM, 8 reps). Three sets of 15 crunches were performed both days, and participants rested one minute between each set and exercise.

The HIFT group utilized CrossFit™ training for 60-minute sessions led by CrossFit™ certified trainers. Nine movements (i.e., air squat, front squat, overhead squat, press, push press push jerk, deadlift, sumo deadlift high pull, and medicine ball clean) were introduced in sessions 1–2. Remaining sessions included warm-up and stretching (10–15 min), instruction and technique practice (10–20 min), workout (5–30 min), and cool-down and stretching (5 min). Workouts utilized aerobic (e.g., rowing), bodyweight (e.g., pushups), and weightlifting (e.g., deadlifts) exercises in singular or multiple combinations that were completed for time, repetitions, or weight (e.g., see Table 1). Workouts were completed at relative (self-selected) high-intensity for each participant, with movements and weights individually scaled.

**Table 1:** **High-intensity functional training workouts for week 4**

Day	Monday	Wednesday	Friday
Workout	2 rounds for time of	3 rounds for max reps of	10 rounds for time of
	-7 muscle-ups	-1 minute right-arm	-8 burpees
	-30 mountain climbers	dumbbell power snatch	-30 bleacher step runs
	-3 overhead squats	-30 seconds rest	
	-6 front squats	-12 minutes front squats	
	-9 back squats	-30 seconds rest	
	-30 mountain climbers	-1 minute left-arm	
	-12 handstand pushups	dumbbell power snatch	
		-30 seconds rest	
Range for time or reps	9:42–18:36	65-174 reps	16:32–23:16
Modifications	-muscle-up transitions with knees on floor	-dumbbell weight (3.6-6.8 kg)	-stepping back (versus jumping) for burpees
	-barbell weight (3.6-29.5 kg)	-barbell weight (6.8-43.1 kg)	-knee pushups for burpees
	-pike pushups using elastic band or feet on box for assistance		-repetitions (6 burpees and 15 bleacher steps)

### Main outcome measures

At baseline, participants were asked to indicate why they were interested in participating in the study (exercise initiation; qualitative). At baseline and posttest, participants were asked to complete a single-item to rate their exercise enjoyment from 1-strongly disagree to 5-strongly agree for the statement, “I enjoy doing exercise” [19]. This measure has been found to have fair test-retest reliability and strong construct validity and to significantly correlate with objective and self-reported physical activity [19]. At posttest, participants were asked if they planned to continue exercising and what type of exercise they planned to do (exercise intentions; qualitative). Adherence was defined as completing 90% of exercise sessions.

In light workout clothing without shoes at baseline and posttest, participants’ height to the nearest 0.5-cm was measured with a SECA® 214 portable stadiometer (Chino, CA) and their weight to the nearest 0.5-pound was measured with a Detecto scale (Webb City, MO). These measurements were used to calculate BMI, after conversion of pounds to kg. Body composition was measured by Lunar Prodigy dual-energy X-ray absorptiometry (DXA) scan (Madison, WI). Times to complete daily workouts (excluding time for warm-up, stretching, skill work, and cool-down) were recorded.

Qualitative data were analyzed using NVivo (version 10). Reasons for exercise initiation were coded as either intrinsic (i.e., focused on the process of completing the behavior such as pleasure, satisfaction, and skill development) or extrinsic (i.e., focused on completing the behavior to gain benefits, improve image, or avoid negative consequences) [19–21]. Categories of qualitative answers were counted and entered into SPSS (version 20) for statistical analysis.

Quantitative data were summarized with descriptive statistics. To examine between-group differences at baseline, independent samples t-tests were conducted for age, BMI, body composition, and exercise enjoyment; chi-square analysis was conducted for gender, marital status, race/ethnicity, education, and income. Paired samples t-tests were used to compare dropouts to adherers. To examine differences from baseline to posttest in exercise enjoyment, BMI, and body composition, 2 (group) × 2 (time) repeated measures ANOVAs (between-groups) were conducted. Chi-square analysis was conducted to compare exercise intentions between groups. Independent samples t-tests were conducted to compare workout times. Statistical significance was set at p < .05.

## Results

Baseline participant characteristics by group are provided in Table 2. The only significant difference was for exercise enjoyment, with ART participants reporting significantly lower exercise enjoyment than HIFT participants, t = 2.1, p = 0.049.

**Table 2:** **Baseline participant characteristics by study group**

Characteristic	ART Group (n = 11)	HIFT Group (n = 12)	p-value
Age	28.0 ± 5.7	28.3 ± 7.1	0.93
% Female	72.7 (8)	41.7 (5)	0.14
% Single	63.6 (7)	66.7 (8)	0.64
Race/Ethnicity %			0.11
Asian	18.2 (2)	-	
Black	9.1 (1)	-	
White	90.9 (7)	75.0 (9)	
Hispanic/Latino	-	25.0 (3)	
Missing	9.1 (1)	-	
Education %			0.19
Some college	27.3 (3)	41.7 (5)	
Bachelor’s degree	36.4 (4)	50.0 (6)	
Master’s degree	27.3 (3)	-	
Doctoral degree	-	8.3 (1)	
Missing	9.1 (1)	-	
Income %			0.48
< $20,000	27.3 (3)	50.0 (6)	
$20,001-$30,000	18.2 (2)	8.3 (1)	
$30,001-$40,000	18.2 (2)	8.3 (1)	
$40,001-$50,000	-	8.3 (1)	
>$50,000	27.3 (3)	25.0 (3)	
Missing	9.1 (1)		
BMI (kg/m^2^)	30.2 ± 3.4	31.9 ± 3.5	0.24
Body fat percentage	43.7 ± 7.2	40.5 ± 7.4	0.31
Exercise enjoyment^a^	3.0 ± 1.2	3.9 ± 0.8	0.049

^a^Range 1 (strongly disagree) to 5 (strongly agree) to the statement “I enjoy doing exercise.”

Twenty-two participants provided reasons for exercise initiation, with many listing multiple reasons. Overall, 9 participants (39.1%) listed only intrinsic reasons and 13 (56.5%) listed both intrinsic and extrinsic reasons. For the ART participants, extrinsic reasons included wanting to lose weight (n = 7) and to work with a trainer (n = 2). Intrinsic reasons included to have motivation to exercise (n = 4), to develop an exercise habit (n = 3), to improve fitness/get into shape (n = 3), to learn new exercises/gain knowledge (n = 3), to lose stress (n = 1), and to learn about nutrition (n = 1). HIFT participants were also extrinsically motivated to participate by wanting to lose weight (n = 5). Intrinsic reasons included to have the motivation to exercise (n = 4), to develop an exercise habit (n = 3), to improve health (n = 3), to see results (n = 3), to improve fitness/get into shape (n = 2), and to be exposed to a new type of exercise (n = 1).

Two participants dropped out of the ART group, one citing scheduling issues and one not providing a reason. Three participants dropped out of the HIFT group, one citing scheduling issues, one having a pulled groin muscle, and one not providing a reason. HIFT dropouts had significantly lower baseline exercise enjoyment than HIFT adherers, M = 3.0 ± 1.0 versus M = 4.2 ± 0.4; t(10) = 3.08, p = .012 (95% CI = .34 to 2.11). The following results are for study adherers only (n = 18).

**Figure 1:**
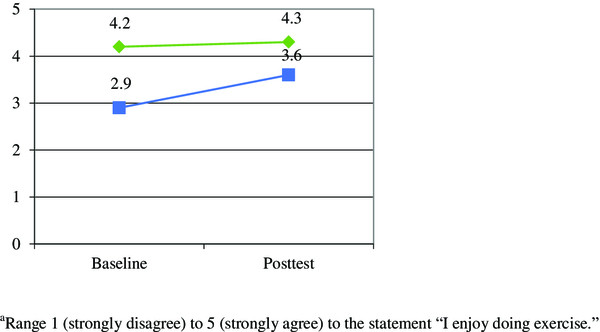
**Group by time interaction for changes in exercise enjoyment** ^**a**^ **.**

The 2x2 RANOVA demonstrated a statistically significant main effect for changes over time in exercise enjoyment [F(1, 16) = 14.52, p = .002]. There was also a main effect between groups [F(1, 16) = 4.97, p = .04], as well as a significant group*time interaction effect [F(1, 16) = 7.81, p = .013] (see Figure 1).

BMI did not significantly change from baseline to posttest for either group. DXA scans showed no significant changes in body fat percentage, lean body mass or fat mass for either group. Weight changes were insignificant for both groups (HIFT range = -2.4 to + 1.7 kg; and ART range = -3.4 to +1.6 kg). No significant main effects or interactions were found between groups for changes over time in BMI, body fat percentage, lean body mass, or fat mass.

All but one ART participant intended to continue exercising. All HIFT participants intended to continue the same exercise as compared to only 55.6% of ART participants, χ^2^(1, n = 16) = 9.35, p = .002. Specifically, all HIFT participants (n = 9) planned to continue doing CrossFit™, with four also planning to do additional cardio (e.g., running, biking, swimming). Five ART participants planned to continue doing “cardio and weights,” and two planned to try CrossFit™. Three ART participants planned to take exercise classes (e.g., aerobics, Pilates), two planned to start running and one planned to train for a Half Ironman.

Time spent completing daily workouts was greater for ART (M = 63.3 ± 3.3 minutes) than HIFT participants (M = 13.3 ± 6.4 min; t(16) = 43.5, p < .001). Average minutes for weekly workouts were significantly greater for ART (M = 189.8 ± 10.0 min) than for HIFT participants (M = 39.3 ± 2.5 min; t(16) = 43.7, p < .001).

## Discussion

This is the first study to assess behavioral responses to HIFT training among physically inactive overweight and obese adults. All participants who indicated the extrinsic reason of losing weight, also stated an intrinsic reason for exercise initiation. HIFT participants with higher baseline exercise enjoyment were significantly more likely to complete the study and were significantly more likely to plan to continue the same exercise than those in the prescribed intensity (ART) condition.

Previous research found improvements in exercise enjoyment for moderate-intensity exercise, similar to those for ART participants [5]. As enjoyment is inversely related to exercise intensity, it is not surprising that those with lower baseline exercise enjoyment dropped out of HIFT [5, 12]. It is possible they might have adhered if they had been in the ART group.

To determine if participant attrition affected our outcome measures, we examined our data using intention to treat analysis, but results did not significantly differ [http://www.consort-statement.org/Contents/Item/Display/500].

Limitations included no measures for intensity or affect changes during HIFT workouts, potentially missing variations in enjoyment that affected intentions [5]. The act of enrolling in an 8-week exercise study where eligibility criteria included being overweight or obese and physically inactive may have biased participants’ self-reported answers for exercise initiation and enjoyment. One participant randomized to the ART condition indicated wanting to try CrossFit™ and losing weight as his reasons for enrolling in the study. This potentially biased for his intention to try CrossFit™ at the conclusion of the study, although he also planned to run a 5-km race. We did not verify whether participants followed through on exercise intentions (although four enrolled in regular CrossFit™ classes with two continuing today). Finally, due to the small sample size, these results may not generalize outside of these participants.

Study strengths included adherence; dropout rates of 19% for ART and 25% for HIFT participants were lower average (45%) for exercise interventions [22]. Only one HIFT participant (4.3%) experienced an injury in our study, indicating that the HIFT protocol was safely conducted among our participants. Future research could include a larger sample with extended follow-up to determine sustained adherence as well as injury rates per 1000 hours (total training time for all HIFT participants was only 52.6 hours) [18].

## Conclusions

Our data have public health implications in that HIFT participants spent significantly less time exercising than both ART participants [6, 7] and current US physical activity guidelines [2], yet were able to maintain exercise enjoyment and intend to continue [9]. This may be due to self-selected intensity levels, exercise variance, or novelty of HIFT. However, it may be helpful for adults with lower exercise enjoyment to initiate moderate-intensity ART training when beginning a new exercise program, as significant improvements in exercise enjoyment can occur. The HIFT training was time-efficient and safely conducted for these previously inactive overweight and obese adults. Participants were able to maintain their body composition during the exercise programs, with some losing over 3 kg. Due to selection of CrossFit™ for HIFT, participants could continue participation almost anywhere [23]. Ultimately, public health researchers and practitioners would be well-served to include time-efficient high-intensity exercise options when promoting physical activity [5].

## Abbreviations

1RM: One repetition maximum
ART: Aerobic and resistance training
BMI: Body mass index
DXA: Dual X-ray absorptiometry
HIFT: High-intensity functional training
HIT: High-intensity training
HRR: Heart rate reserve
US: United States.
